# In Situ Hydrogel Modulates cDC1‐Based Antigen Presentation and Cancer Stemness to Enhance Cancer Vaccine Efficiency

**DOI:** 10.1002/advs.202305832

**Published:** 2024-04-02

**Authors:** Tong Gao, Shijun Yuan, Shuang Liang, Xinyan Huang, Jinhu Liu, Panpan Gu, Shunli Fu, Na Zhang, Yongjun Liu

**Affiliations:** ^1^ Department of Pharmaceutics Key Laboratory of Chemical Biology (Ministry of Education) NMPA Key Laboratory for Technology Research and Evaluation of Drug Products School of Pharmaceutical Sciences Cheeloo College of Medicine Shandong University 44 Wenhua Xi Road Jinan Shandong 250012 China

**Keywords:** cancer combination therapy, cancer stemness, cDC1 vaccine, immunogenic cell death, in suit vaccine

## Abstract

Effective presentation of antigens by dendritic cells (DC) is essential for achieving a robust cytotoxic T lymphocytes (CTLs) response, in which cDC1 is the key DC subtype for high‐performance activation of CTLs. However, low cDC1 proportion, complex process, and high cost severely hindered cDC1 generation and application. Herein, the study proposes an in situ cDC1 recruitment and activation strategy with simultaneous inhibiting cancer stemness for inducing robust CTL responses and enhancing the anti‐tumor effect. Fms‐like tyrosine kinase 3 ligand (FLT3L), Poly I:C, and Nap‐CUM (NCUM), playing the role of cDC1 recruitment, cDC1 activation, inducing antigen release and decreasing tumor cell stemness, respectively, are co‐encapsulated in an in situ hydrogel vaccine (FP/NCUM‐Gel). FP/NCUM‐Gel is gelated in situ after intra‐tumoral injection. With the near‐infrared irradiation, tumor cell immunogenic cell death occurred, tumor antigens and immunogenic signals are released in situ. cDC1 is recruited to tumor tissue and activated for antigen cross‐presentation, followed by migrating to lymph nodes and activating CTLs. Furthermore, tumor cell stemness are inhibited by napabucasin, which can help CTLs to achieve comprehensive tumor killing. Collectively, the proposed strategy of cDC1 in situ recruitment and activation combined with stemness inhibition provides great immune response and anti‐tumor potential, providing new ideas for clinical tumor vaccine design.

## Graphical abstract

An in situ hydrogel vaccine is fabricated by co‐encapsulating Fms‐like tyrosine kinase 3 ligand (FLT3L), Poly I:C, and Nap‐Ce6/UCM (NCUM). Ce6 triggered Photodynamic therapy (PDT) induces tumor antigens release in situ. cDC1 is recruited to tumor tissue and activated for cross‐presentation, followed by migrating to lymph nodes and activating CTLs. Tumor stemness is inhibited by napabucasin, which helps CTLs to achieve comprehensive tumor killing.

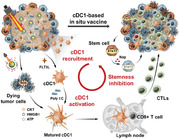

## 1 Introduction

Tumor immunotherapy is currently the most promising strategy for cancer treatment, and among them, therapeutic tumor vaccines are highly preferred.^[^ ^1^ ^]^ More than 300 therapeutic tumor vaccines have been tested in clinical trials, but their clinical outcomes remain unsatisfactory.^[^ ^2^ ^]^ Insufficient priming and limited quantity of cytotoxic T lymphocytes (CTLs), which are the central effector cells in immune response, are the main reasons for poor vaccine efficacy.^[^ ^3^ ^]^ Thereby, how to induce an effective tumor‐specific CTLs response has been a hot spot in the field of tumor vaccine.

Dendritic cells (DCs) play a core role in initiating and maintaining tumor‐specific CTLs immune responses. The efficacy of therapeutic tumor vaccines is highly dependent on the antigen processing and antigen presentation efficiency of DCs to effector T cells.^[^ ^4^ ^]^ Recent studies have highlighted that different DC subtypes determine their efficacy in T cell priming.^[^ ^5^ ^]^ DCs are classified as monocyte‐derived DC (MoDC), plasmacytoid DCs (pDC), type 1 conventional DCs (cDC1), and type 2 conventional DCs (cDC2). At the functional level, DC subpopulations are specialized to produce different responses to different pathogens.^[^ ^6^ ^]^ MoDC mainly plays a role in inflammation and infection. pDC specializes in the production of type I interferon (IFN), however, the ability of pDC to produce IFN in cancer appears to be impaired. cDC2 controls the response to extracellular pathogens by presenting antigens to helper CD4 T cells. cDC1, a special subpopulation of DCs with antigen natural cross‐presentation characteristic, can selectively and effectively induce antigen‐specific CTLs by phagocytosis, processing, and exposure of exogenous antigen fragments, which has a direct benefit to the anti‐tumor immune response.^[^ ^7^ ^]^ However, the clinical used DC subtypes for cancer vaccines are mainly MoDC, pDC, and cDC2 subtypes.^[^ ^8^ ^]^ MoDCs are not equipped with full co‐stimulatory molecules and antigen cross‐presentation mechanisms and MoDC function is susceptible to be inhibited by the tumor microenvironment thus limiting their efficacy,^[^ ^4a^ ^]^ pDC and cDC2 are scarce in body and high‐cost.^[^ ^9^ ^]^ In situ recruitment of DCs using granulocyte‐macrophage colony‐stimulating factor (GM‐CSF) has been widely reported,^[^ ^10^ ^]^ which recruit mainly MoDC subtype.^[^ ^11^ ^]^ Therefore, vaccine efficiency may be improved by exploiting a more appropriate DC subtype, such as cDC1.

The use of in vitro‐generated cDC1 as a tumor vaccine has been investigated recently. Zhou et al. has demonstrated that cDC1 obtained from bone marrow are significantly more effective than MoDC.^[^ ^12^ ^]^ However, this in vitro cDC1 generation strategy faces the problems of low cDC1 proportion, complex process, and high cost, making cDC1 in situ recruitment a more promising strategy. The differentiation and recruitment of cDC1 are regulated by Fms‐like tyrosine kinase 3 ligand (FLT3L).^[^ ^13^ ^]^ A previous clinical trial applied FLT3L to recruit cDC1 in situ, however, no obvious clinical benefit was obtained, which may be caused by the lack of DC activation.^[^ ^14^ ^]^ Thus, the present study proposed a strategy of in situ recruitment and activation of cDC1 simultaneously to strengthen immune response for the first time. Toll‐like receptors (TLRs) recognize pathogen‐associated molecular patterns and play a key role in immune cell regulation, survival, and proliferation.^[^ ^15^ ^]^ TLR3 is an important target that regulates cDC1 activation. We hypothesis that intra‐tumoral administration of FLT3L and Poly I:C (TLR3 agonist) can effectively recruit and activate tumor infiltrating cDC1.

Otherwise, Cancer stem cells (CSCs) are considered to be the source of tumorigenesis, characterized by self‐renewal and heterogeneity, and able to evade recognition and killing by CTLs.^[^ ^16^ ^]^ Despite cancer vaccine‐induced CTLs successfully eliminate the majority of tumor cells, the remaining CSCs are sufficient to drive cancer recurrence.^[^ ^17^ ^]^ Signal transduction and activator of transcription 3 (STAT3) is a key factor in maintaining the stemness of tumor cells.^[^ ^18^ ^]^ Napabucasin, as a STAT3 inhibitor, can effectively inhibit the transcription of genes driven by STAT3, showing a significant ability to reduce the stemness of tumor cells.^[^ ^19^ ^]^ Therefore, the combination of cancer vaccine and napabucasin can promote the CTLs to effectively kill conventional tumor cells and heterogeneous tumor cells, so as to more efficiently eliminate the tumor.

**Scheme 1:**
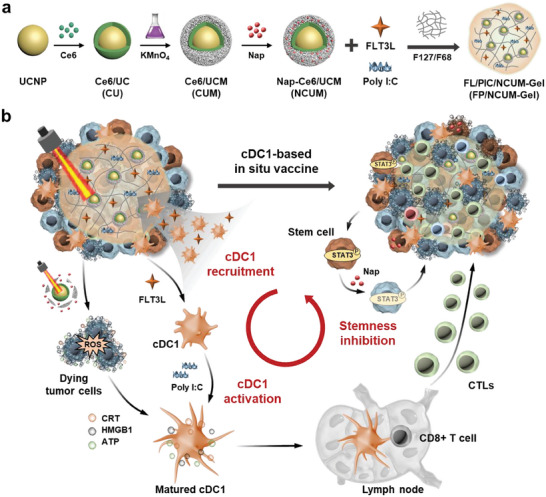
Schematic illustration of cDC1‐based intrinsic vaccination strategy to improve the efficacy of cancer vaccine. a) Preparation of FP/NCUM‐Gel. b) FP/NCUM‐Gel induced immunogenic cell death of tumor cells, recruited cDC1 to cross‐presentation tumor antigens, activated CTLs immune response specifically, and reduced tumor stemness to enhance tumor killing effect.

Herein, a combination strategy based on cDC1 recruitment was proposed (**Scheme** **1**). Photodynamic therapy (PDT), which have been widely studied as a promising vaccination strategy,^[^ ^20^ ^]^ was used to induce in situ antigen release. Meanwhile, intra‐tumoral injection of cytokine FLT3L and TLR3 agonist Poly I:C to recruit and activate cDC1, promote antigen cross‐presentation and specifically activate CTLs immune response. In addition, STAT3 inhibitor napabucasin was employed to reduce the stemness of tumor cells, thus allowing CTLs to eliminate tumor efficiently. In order to achieve the design of in situ vaccine induction and co‐delivery of drugs with different properties, the photosensitizer chlorin e6 (Ce6) for PDT was modified on the surface of the up‐conversion nanoparticles to allow long wavelength triggered deep tumor PDT, and then coated with manganese dioxide to obtain CUM (CUM). Napabucasin was physically encased in the manganese dioxide layer to obtain Nap‐Ce6/UCM (NCUM). FLT3L, Poly I:C and NCUM were co‐loaded in thermosensitive hydrogel to form FP/NCUM‐Gel as a drug depot. In general, FP/NCUM‐Gel, after intra‐tumoral injection, can recruit and activate cDC1 to cross‐presentation tumor antigens and stimulate CTLs immune response continuously. Further, reducing the stemness of tumor cells with napabucasin can facilitate CTL triggered all‐round tumor killing and improve cancer therapeutic effect. Overall, this study provides a promising cDC1‐based in situ vaccine for improving therapeutic cancer vaccine efficiency.

## 2 Results and Discussion

### 2.1 Preparation and Characterization of NCUM

**Figure 1:**
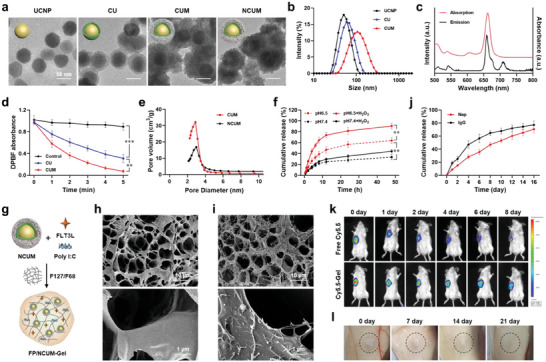
Characterization of FP/NCUM‐Gel. a) TEM images and b) hydrodynamic size of UCNP, CU, CUM and NCUM. c) Fluorescence emission spectra and UV–vis absorption spectra of CUM. d) Absorption of DPBF mixed with H_2_O, CU, and CUM upon 980 nm laser irradiation for different time durations. e) Pore size distribution of CUM and NCUM. f) Release profiles of Nap from NCUM incubated in PBS with or without H_2_O_2_ (1 mm). g) Schematic diagram of the hydrogel for the encapsulation of FLT3L, Poly I:C, and NCUM. SEM images of h) blank gel and i) FP/NCUM‐Gel. j) Release profiles of Nap and IgG from hydrogels. k) Drug retention behavior of free Cy5.5 and Cy5.5‐gel in vivo. l) Degradation behavior of the gel in vivo within 21 days.

CUM was prepared using a layer‐by‐layer assembly method (Figure S1, Supporting Information). First, Ce6 was modified with amino‐functionalized silane APTES and then immobilized onto the surface of the up‐conversion nanoparticles (UCNP) through silane dehydration condensation to obtain Ce6/UC (CU). Subsequently, a layer of manganese dioxide (MnO_2_) was coated on the surface of CU to obtain CUM. Transmission electron microscopy (TEM) images showed that UC NPs was a spherical nanoparticle with a smooth surface. A thin layer of silica was observed on the surface of CU, and a thicker layer of MnO_2_ was observed on the surface of CUM (**Figure** **1**a). The particle sizes of UCNP, CU, and CUM were 53.82 ± 6.97 nm, 67.84 ± 1.13 nm, and 109.83 ± 7.27 nm, respectively (Figure 1b; Table S1, Supporting Information). The corresponding zeta potentials were 16.47 ± 2.21 mV, −7.14 ± 0.68 mV, and −21.37 ± 2.10 mV, respectively (Figure S2a, Table S1, Supporting Information). Ce6 was immobilized in the silica layer, and the drug loading and encapsulation efficiency were 4.06 ± 0.21% and 87.49 ± 1.11%, respectively (Table S2, Supporting Information). Overall, these results demonstrate the successful preparation of CUM.

The fluorescence emission and UV–vis absorption spectra of CUM showed that under an excitation wavelength of 980 nm, CUM exhibited characteristic emission peaks at 630–680 nm, which matched the absorption peaks of CUM (Figure 1c). This indicated that under 980 nm laser irradiation, CUM could convert long‐wavelength light to shorter‐wavelength light, thereby activating the photosensitizer Ce6 to produce photodynamic therapeutic effects. The ability of CUM to promote reactive oxygen species (ROS) generation in vitro was evaluated using DPBF assay. After irradiation with a 980 nm near‐infrared laser at a power density of 0.5 W cm^−2^, there was no significant change in absorbance of the DPBF solution (Figure 1d; Figure S2c, Supporting Information). The absorbance of the DPBF and CUmixed solution significantly decreased after laser irradiation (*p* < 0.001) (Figure S2d, Supporting Information), indicating that CU could generate ROS upon laser irradiation to consume DPBF. The absorbance of the DPBF and CUM mixed solution after laser irradiation was significantly lower than that of the DPBF and CUmixed solution (*p* < 0.01) (Figure S2e, Supporting Information), possibly due to the degradation of the MnO_2_ layer under acidic conditions to generate O_2_, which promotes the photochemical reaction to produce ROS. These results indicate that CUM can generate ROS for photodynamic therapy under 980 nm near‐infrared laser irradiation.

By passive loading method, Napabucasin was loaded into the pores of MnO_2_ layer to obtain NCUM. The drug loading and encapsulation efficiency of Napabucasin in NCUM were 9.58 ± 0.57% and 42.55 ± 3.03%, respectively (Table S2, Supporting Information). After Napabucasin loaded, the pore volume of NCUM decreased significantly compared to CUM (Figure 1e, Figure S2b, Supporting Information), indicating effective drug loading. Furthermore, MnO_2_ can be degraded to Mn^2+^ and O_2_ under acidic and redox conditions. The release characteristics of Napabucasin were evaluated by incubating NCUM at different pH values with H_2_O_2_.The results showed that the release of Napabucasin from NCUM was 33.31 ± 4.85% at pH 7.4, and significantly increased to 63.89 ± 5.54% at pH 6.5 (*p* < 0.01) within 48 h (Figure 1f). And adding H_2_O_2_ to the release medium at pH 6.5 increased the release of Napabucasin to 90.55 ± 5.35%, significantly higher than the group without H_2_O_2_ (*p* < 0.01). These results indicate that Napabucasin encapsulated in the MnO_2_ layer has pH and redox‐sensitive release characteristics.

### 2.2 Preparation and Characterization of FP/NCUM‐Gel

Poloxamer is a classic thermosensitive gel material, characterized by low toxicity, high biocompatibility, and stable chemical properties. By adjusting the concentration and proportion of its homologs, it can undergo gelation at specific temperatures. The gelation temperature decreases with the of increasing F127 concentration, while it increases with increasing F68 concentration. When the F127 concentration is 18% and the F68 concentration is 4%, the gelation temperature is 35 °C (Table S3, Supporting Information), which meets the requirements for in situ gel formation after intratumoral injection (Figure S3, Supporting Information). Under this temperature, the gelation time is 82 s, and its injectability is good. Scanning electron microscopy (SEM) reveals that the structure of the gel is highly porous and interconnected, with pore sizes ranging from 2 to 10 µm (Figure 1h). NCUM, FLT3L, and Poly I:C were added to the F127/F68 solution to prepare a drug‐loaded hydrogel, which exhibited similar structural features to the blank hydrogel under scanning electron microscopy (Figure 1g). Furthermore, small particle‐like protrusions were observed on the surface of the gel (Figure 1i). The gelation temperature and time of the drug‐loaded hydrogel did not differ significantly from those of the blank hydrogel.

Subsequently, the drug release behavior of the hydrogel was investigated. NCUM and model protein IgG were loaded into the hydrogel. Both Napabucasin and IgG exhibited sustained release properties, with release amounts of 70.56 ± 5.39% and 77.18 ± 5.85%, respectively, within 16 days (Figure 1j). IgG was released faster than Napabucasin, possibly because IgG was passively diffused from the gel, while Napabucasin was loaded into nanoparticles with restricted diffusion. The retention of the drug‐loaded hydrogel in tumor tissue was investigated in CT26 tumor‐bearing mice by using Cy5.5 as a tracer to prepare Cy5.5‐Gel. Real‐time in vivo imaging showed that the fluorescence signal of free Cy5.5 rapidly decreased at the tumor site within 4 days, while the fluorescence signal of Cy5.5‐Gel remained above background for at least 8 days (Figure 1k). The fluorescence signal at the tumor site gradually decreased but could still be detected up to 20 days, indicating good sustained release properties of the hydrogel (Figure S4, Supporting Information). Gelation and degradation behavior of the hydrogel in vivo were investigated in Balb/c mice by subcutaneously injecting the hydrogel. At the predetermined time points, mice were euthanized and dissected, and the results showed that the hydrogel gelled after injection and gradually degraded within 21 days (Figure 1l).

### 2.3 ROS Generation and Cell Apoptosis Induced by NCUM

**Figure 2:**
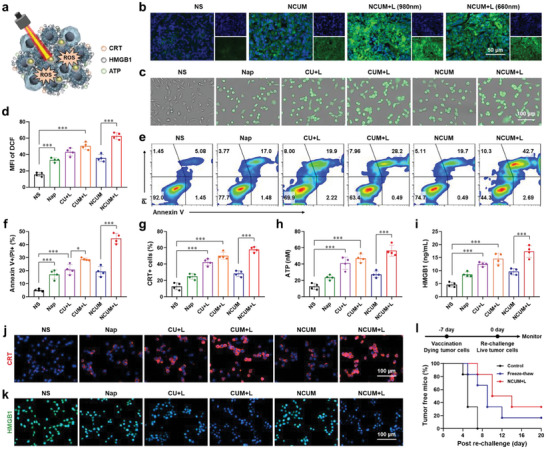
NCUM induced immunogenic cell death (ICD) of tumor cells and produced vaccine‐like effects. a) Schematic diagram of ICD triggered by NCUM. b) Fluorescence microscope images of ROS production in CT26 tumors. c) Fluorescent images and d) flow cytometry analysis of ROS production in vitro. e,f) Flow cytometry analysis of CT26 cells apoptosis. g) Flow cytometry analysis of CRT expression in vitro. h) ELISA analysis of HMGB1. i) ECL analysis of ATP secretion. j) Immunofluorescence analysis of CRT and k) HMGB1 in vitro. l) Treatment schedule for antitumor vaccination and the percent of tumor‐free mice vaccinated with dying CT26 cells pretreated with freeze‐thaw process and NCUM+L.

The ability of NCUM to induce ROS production in vivo was investigated (**Figure** **2**a). The fluorescent imaging of tumor tissue sections showed that after irradiation with 980 nm and 660 nm near‐infrared lasers, ROS generation in tumor tissue increased, but the ROS content in 980 nm group was higher than 660 nm group, possibly due to the stronger tissue penetration ability of the 980 nm laser (Figure 2b). To investigate the photodynamic therapy capability of NCUM at the cellular level, the generation of ROS on CT26 cells was determined. The fluorescence microscopy images and flow cytometry results showed that the intracellular ROS content in the CUM+L group was 50.43 ± 4.46%, significantly higher than that in the NS group 15.63 ± 2.33% (*p* < 0.001) (Figure 2c,d). The intracellular ROS of NCUM+L group was 62.58 ± 4.09%, significantly higher than NCUM group 35.60 ± 4.37%. These results implied that NCUM can significantly promote ROS production in tumor tissue and cells under 980 nm near‐infrared laser irradiation.

Subsequently, the apoptosis‐inducing capability of NCUM on CT26 cell was evaluated. Flow cytometry analysis showed that the apoptosis rate of the Napabucasin group was 17.18 ± 3.51%, while that of the NS group was only 5.05 ± 0.94% (*p* < 0.001), indicating that Napabucasin had a pro‐apoptotic effect (Figure 2e,f). The NCUM+L group exhibited the highest apoptosis‐inducing potential, indicating that photodynamic therapy could effectively promote cell apoptosis. The CUM+L group had an apoptosis rate of 28.73 ± 0.97%, significantly higher than the CU+L group, demostrating that the presence of the MnO_2_ layer could enhance the effectiveness of photodynamic therapy. The above results demonstrate that NCUM can effectively promote tumor cell apoptosis in vitro.

### 2.4 Immunogenic Cell Death Induced by NCUM

Photodynamic therapy promotes the production of ROS, which triggers cellular oxidative stress and inflammation, leading to the release of CRT, HMGB1, and ATP.^[^ ^21^ ^]^ To investigate the ability of NCUM to induce immunogenic cell death, CRT expression on CT26 cells, as well as HMGB1 location and ATP production, were measured. Fluorescence microscopy and flow cytometry results showed that the expression of CRT in CUM+L group was 50.25 ± 4.40%, significantly higher than NS group (12.85 ± 4.27%) (Figure 2g,j). The NCUM+L group showed the highest intensity of intracellular red fluorescence, and the expression of CRT was 57.93 ± 3.30%. Furthermore, the intracellular HMGB1 levels were detected. The fluorescence of HMGB1 in the nucleus of CU+L, CUM+L, and NCUM+L groups was reduced (Figure 2k), but the corresponding HMGB1 levels in the cell culture supernatant were increased, which were 12.47 ± 0.78 ng mL^−1^, 14.59 ± 2.14 ng mL^−1^, and 17.33 ± 2.30 ng mL^−1^, respectively (Figure 2h), indicating that laser irradiation significantly promotes the release of intracellular HMGB1. The release of ATP also showed a similar trend (Figure 2i). The secretion of ATP in CU+L and CUM+L groups increased to 41.10 ± 7.53 nm and 47.16 ± 5.00 nm, respectively. The secretion of ATP in NCUM+L group increased to 56.45 ± 6.76 nm. All of the above results consistently demonstrate that NCUM can effectively induce immunogenic cell death in vitro.

The immune response following tumor cell death induced by immunogenic cell death was investigated. Classic freeze‐thaw treated tumor cells were used as a control, while CT26 cells pre‐treated with PBS and NCUM+L were subcutaneously injected in the inguinal region. After 7 days, normal tumor cells were injected into the axillary. The results showed that on the 7th day after the injection of normal tumor cells, the tumor‐free rate was 0% in the NS group, 66.67% in the freeze‐thaw group, and 83.33% in the NCUM+L group (Figure 2l). This indicated that the injection of freeze‐thaw pre‐treated and NCUM+L pre‐treated CT26 cells could delay or inhibit the growth of tumors, with NCUM+L pre‐treatment showing a stronger effect. This may be due to the freeze‐thaw cycle and NCUM+L promoted release of relevant antigens and immunogenic signals from tumor cells. After subcutaneous injection, they are taken up and processed by DC cells in the body, triggering an immune response. When tumor cells were injected again after 7 days, the growth of the tumor was suppressed by the immune system.

### 2.5 Promoting the Maturation of GM‐DC and FL‐DC In Vitro

**Figure 3:**
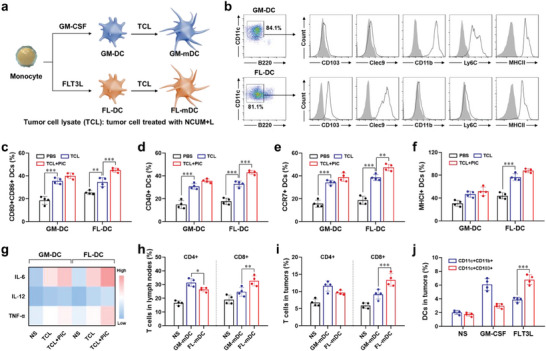
cDC1 stimulated by NCUM enhances the activation of CD8^+^ T cells. a) Schematic diagram of DC induction and maturation stimulated by tumor cell lysate. b) Flow cytometry analysis of CD103, Clec9, CD11b, Ly6C and MHC II expression on GM‐DC and FL‐DC. c) Flow cytometry analysis of CD80 and CD86 expression on DC. d) Flow cytometry analysis of CD40 expression on DC. e) Flow cytometry analysis of CCR7 expression on DC. f) Flow cytometry analysis of MHC I expression on DC. g) Levels of IL‐6, IL‐12, and TNF‐α in the supernatant of DCs. h) Flow cytometry analysis of CD4^+^ and CD8^+^ T cells in lymph nodes. i) Flow cytometry analysis of CD4^+^ and CD8^+^ T cells in tumor tissues. j) Flow cytometry analysis of DC in tumor tissues after intratumoral injection of GM‐CSF or FLT3L. Data were expressed as mean ± SD (*n* = 4).

GM‐DC were generated from mononuclear cells derived from Balb/c mouse bone marrow by induction with GM‐CSF (**Figure** **3**a). Flow cytometry analysis revealed that the proportion of induced CD11c^+^B220^−^ DC cells was 84.1%, which expressed high levels of the hallmark molecules CD11b, Ly6C, and MHC II of the MoDC subset (Figure 3b). FL‐DC were generated from mononuclear cells derived from Balb/c mouse bone marrow by induction with FLT3L (Figure 3a). Flow cytometry revealed that the proportion of induced CD11c^+^B220^−^ DC cells was 81.1%, which expressed high levels of the hallmark molecules CD103, Clec9, and MHC II of the cDC1 subset (Figure 3b).

DC plays a key role in initiating the CTL response. NCUM induces ICD effects in tumor cells, releasing DAMPs that promote DC maturation. GM‐DC and FL‐DC were separately co‐cultured in vitro with NCUM pre‐treated CT26 cells and adjuvant Poly I:C, and their maturation and molecular expression were observed. Flow cytometry analysis showed that after co‐cultured with NCUM pre‐treated CT26 cells, the mature GM‐DC and FL‐DC content was 35.58 ± 2.56% and 34.58 ± 3.89%, respectively, which were significantly higher than PBS group (Figure 3c). The addition of Poly I:C further promoted FL‐DC maturation, increasing the content to 45.05 ± 1.84% (*p* < 0.001), while Poly I:C had no significant effect on GM‐DC maturation (*p* > 0.05). This may be because FL‐DC has the characteristics of cDC1 cells, which can express high levels of TLR3 receptors, while GM‐DC has the characteristics of MoDC cells with lower TLR3 expression. GM‐DC and FL‐DC also exhibited similar trends in the expression of surface molecules CD40 and CCR7 (Figure 3d,e). These results indicated that tumor cells treated with NCUM could promote DC maturation and enhance their homing ability in vitro. Subsequently, the surface expression of MHC I molecules was evaluated, and the flow cytometry analysis showed that after co‐culture with NCUM pre‐treated CT26 cells, MHC I was significantly upregulated in FL‐DC (*p* < 0.001), while the change in GM‐DC was not obvious, indicating that FL‐DC cells could present tumor antigens through MHC I molecules and have stronger cross‐presentation ability (Figure 3f). Furthermore, the cytokine levels in the supernatant of DC cultures were determined, and the content of IL‐6, IL‐12, and TNF‐α were significantly increased after co‐culture of DCs with NCUM pre‐treated CT26 cells (Figure 3g). These results indicate that NCUM pre‐treated CT26 cells can effectively stimulate the maturation of GM‐DC and FL‐DC, promote the expression of surface molecules, and stimulate cytokine secretion.

Matured DC cells presents antigens to T cells, promoting their proliferation and activation into effector T cells, thereby exerting antitumor immune effects. GM‐DC and FL‐DC, when co‐cultured with NCUM pre‐treated CT26 cells and adjuvant Poly I:C, both matured, accompanied by increased expression of surface molecules, secretion of cytokines, and enhanced homing ability. The difference lies in the fact that FL‐DC can present tumor antigens more through MHC I molecules. Matured GM‐DC (GM‐mDC) and matured FL‐DC (FL‐mDC) were separately injected into tumor‐bearing mice, and the content of T cells in the lymph nodes was collected and analyzed after 7 d. The flow cytometry analysis showed that the content of CD8^+^ T cells of FL‐mDC group was 32.70 ± 3.67%, significantly higher than GM‐mDC group, which was 24.78 ± 3.26% (*p* < 0.01) (Figure 3h). The CD8^+^ T cells infiltrating tumor tissues also showed the same trend, with the FL‐mDC group being 13.38 ± 1.88%, significantly higher than the GM‐mDC group, which was 9.18 ± 1.40% (*p* < 0.001) (Figure 3i). These results indicate that FL‐DC has cDC1 cell characteristics, can cross‐present antigens, and activate more CD8^+^ T cells.

### 2.6 Tumor In Situ Vaccine Induced by FP/NCUM‐Gel

In this study, the ability of intratumoral injection of GM‐CSF and FLT3L to recruit DCs was investigated. The flow cytometry analysis (Figure 3j) showed that the proportion of CD11b^+^ DCs and CD103^+^ DCs in the NS group were 2.02 ± 0.28% and 1.73 ± 0.27%, respectively. After intratumoral injection of GM‐CSF, the content of CD11b^+^ DCs increased to 6.07 ± 0.85% (*p* < 0.001), while after intratumoral injection of FLT3L, the content of CD103^+^ DCs increased to 6.78 ± 0.70% (*p* < 0.001). Compared with GM‐CSF, FLT3L increased the proportion of CD103^+^ DCs in tumor tissue (*p* < 0.001) (Figure S5, Supporting Information), which facilitates the in situ uptake of tumor antigens and cross‐presentation to activate CD8^+^ T cell response. Therefore, FLT3L, Poly I:C, and NCUM were co‐loaded in a thermosensitive hydrogel to obtain FP/NCUM‐Gel. Upon intratumoral injection, FP/NCUM‐Gel formed an in situ drug depot, which could induce an in situ vaccine effect under the laser irradiation.

**Figure 4:**
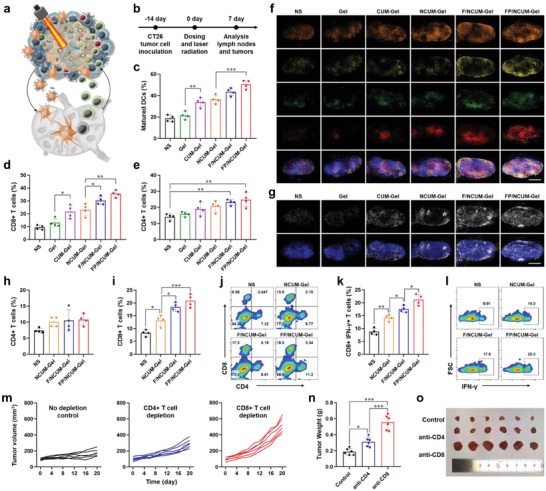
FP/NCUM‐Gel induced in situ vaccine effect and enhanced CD8^+^ T immune response. a) Schematic diagram of DCs and T cells migrating between tumor tissue and lymph nodes. b) Treatment and analysis schedule for CT26 tumor‐bearing mice. c) Flow cytometry analysis of matured DC in lymph nodes. d,e) Flow cytometry analysis of CD8^+^ and CD4^+^ T cells in lymph nodes (gated on CD3^+^ cells). f) Immunofluorescence analysis of lymph node sections at day 7 postvaccination. g) Immunofluorescence analysis of Ki67 expression in lymph nodes. h–j) The proportion of CD4^+^ and CD8^+^ T cells in tumor tissues. k,l) Flow cytometry analysis of CD8^+^IFN‐γ^+^ T cells in tumor tissues. m) Individual tumor growth curves after intratumor implantation of FP/NCUM‐Gel with or without intraperitoneal injection of anti‐mouse CD4 or anti‐mouse CD8α. n,o) Tumor weight and tumor picture.

DCs uptake antigens in tumor tissues, mature, and migrate to lymph nodes to achieve the activation and proliferation of T cells, thus exerting an antitumor immune response (**Figure** **4**a). To investigate the ability of FP/NCUM‐Gel to promote DC maturation after intratumoral injection, lymph node immune cells were collected to analyze the content of mature DCs. Flow cytometry analysis results (Figure 4c; Figure S6a, Supporting Information) showed that the content of mature DCs in the NS group was 18.60 ± 3.03%, while the Blank Gel group was 21.40 ± 3.50%, with no significant difference. The content of mature DCs in the CUM‐Gel group was 33.68 ± 4.26%, which was notablely higher than the Gel group (*p* < 0.01), due to the vaccine‐like effect triggered by CUM on tumor cells, promoting DC maturation. With the addition of FLT3L and Poly I:C, DC maturation was further increased, and the content of mature DCs in the FP/NCUM‐Gel group was the highest, at 50.35 ± 3.96%. Above results illustrated that intratumoral injection of FP/NCUM‐Gel followed by laser irradiation can effectively promote lymph node DC maturation.

Mature DCs in lymph nodes can activate of T cells into effector T cells. Lymph node immune cells were collected to analyze the proportion of CD3^+^CD8^+^ T cells and CD3^+^CD4^+^ T cells. The content of CD3^+^CD8^+^ T cells in CUM‐Gel group was 21.53 ± 5.08%, higher than that Gel group (Figure 4d; Figure S6b, Supporting Information). The CD3^+^CD8^+^ T cells content in the F/NCUM‐Gel and FP/NCUM‐Gel groups were 30.18 ± 3.24% and 35.50 ± 2.61%, respectively, both significantly higher than NUCM‐Gel group. The F/NCUM‐Gel and FP/NCUM‐Gel groups also showed a high level of CD3^+^CD4^+^ T cells content, at 23.13 ± 1.74% and 24.68 ± 4.34%, respectively (Figure 4e). Furthermore, the content of CD8^+^CD69^+^ T cells had the same trend as that of CD3^+^CD8^+^ T cells, with the FP/NCUM‐Gel group having the highest content of CD8^+^CD69^+^ T cells, at 26.93 ± 1.11% (Figure S7, Supporting Information). These results suggest that intratumoral injection of FP/NCUM‐Gel can effectively promote the activation and proliferation of T cells in lymph nodes. Especially, the content and activity of CD8^+^ T cells increased significantly after adding FLT3L and Poly I:C, which may be attributed to the recruitment of cDC1 to the tumor tissue by FLT3L and the stimulation of cDC1 maturation by Poly I:C, thus promoting the cross‐presentation of tumor antigens.

Furthermore, lymph node slices were prepared for immunofluorescence staining analysis, and the results showed that the CUM‐containing group exhibited enhanced orange fluorescence of CD11c, which was induced by CUM‐mediated immunogenic cell death of tumor cells, promoting DC cell maturation, and accompanied by enhanced green fluorescence of CD4 and red fluorescence of CD8, indicating effective activation of T cells (Figure 4f). Upon addition of FLT3L and Poly I:C, significant yellow fluorescence of CD103 was observed, along with significantly higher red fluorescence of CD8 than in other groups, which was attributed to the recruitment and activation of cDC1 by FLT3L and Poly I:C, leading to stronger CD8^+^ T cell activation and proliferation. Furthermore, Ki67 staining was performed on the lymph nodes to examine the cell proliferation ability, and the F/NCUM‐Gel group and FP/NCUM‐Gel group showed more white fluorescence of Ki67, which possibly owing to the increased activation and proliferation of T cells in the lymph nodes (Figure 4g). These results indicate that the injection of FP/NCUM‐Gel into the tumor can recruit and stimulate cDC1 maturation, migration to lymph nodes, and promote the activation and proliferation of CD8^+^ T cells to exert antitumor immune responses.

### 2.7 CD8^+^ T Cells Mediated Antitumor Immunity Enhanced by FP/NCUM‐Gel

The activated effector T cells migrate to tumor site to kill tumor cells. In CT26 tumor‐bearing mouse model, tumor tissues were isolated on the 7th day after treatment, and CD3^+^CD4^+^ T cells and CD3^+^CD8^+^ T cells proportion were analyzed. The results showed a slight change in CD4^+^ T cell content, but a more significant increase was observed in CD8^+^ T cells (Figure 4h–j). The proportion of CD8^+^ T cells in the NCUM‐Gel group was increased to 13.20 ± 2.02%, which may be due to the induction of immunogenic cell death by photodynamic therapy. The percentages of CD8^+^ T cells in the F/NCUM‐Gel group and FP/NCUM‐Gel group grew to 18.40 ± 1.69% and 20.83 ± 2.46%, respectively, indicating that FLT3L and Poly I:C can effectively promote CD8^+^ T cell infiltration into tumor tissue by recruiting and activating cDC1. Furthermore, analysis of CTLs in tumor tissue showed similar results to CD8^+^ T cells (Figure 4k,l). These results suggest that intratumoral injection of FP/NCUM‐Gel can effectively enhance CTL infiltration into tumor tissue by recruiting and activating cDC1 to initiate T cell immune responses.

The direct impact of CD4^+^ and CD8^+^ T cells on tumor progression was evaluated by administrating antibodies of each cell type. The tumor volume of the FP/NCUM‐Gel group was significantly increased from 179.80 ± 42.02 mm^3^ to 297.56 ± 53.47 mm^3^ when CD4^+^ T cells were depleted and to 520.87 ± 93.23 mm^3^ when CD8^+^ T cells were depleted (Figure 4m). Both CD4^+^ and CD8^+^ T cells can affect the antitumor effect of FP/NCUM‐Gel, but the impact of CD8^+^ T cells is more significant (*p* < 0.001) (Figure 4n,o). These findings suggest that the immune‐stimulating ability of FP/NCUM‐Gel relies more on CD8^+^ T cells.

### 2.8 In Vitro Cytotoxicity of NCUM

**Figure 5:**
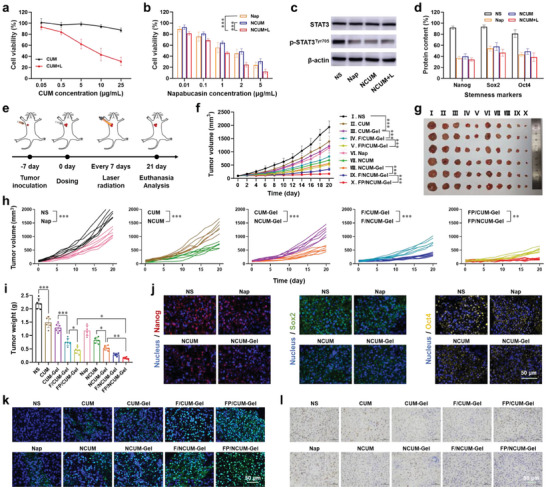
Inhibiting the stemness of tumor cells could further enhance the therapeutic effect of in situ vaccine. a) CT26 viability after treated with CUM and CUM+L. b) CT26 viability after treated with Nap, NCUM and NCUM+L. c) Western blotting of STAT3 and p‐STAT3 expression. d) Flow cytometry analysis of Nanog, Sox2, and Oct4 expression. e) Schedule diagram of administration profile (5 min of 0.6 W/cm^2^ 980 nm laser for one irradiation). f) Average and h) individual tumor volume curves after treated with different formulations. g) Tumor photos and i) tumor weight at the endpoint. j) Immunofluorescence analysis of Nanog, Sox2 and Oct4 expression in CT26 tumors. k) Immunohistochemical analysis of TUNEL in CT26 tumors. l) Immunohistochemical analysis of Ki67 in CT26 tumors.

In vitro cytotoxicity of NCUM was evaluated using the MTT assay. First, the photodynamic toxicity of CUM was examined (980 nm, 0.5 W cm^−2^). The tumor cell survival rate after irradiation was ≈30%, while the survival rate of cells without laser irradiation was ≈100% (**Figure** **5**a). Subsequently, the in vitro cytotoxicity of Napabucasin, NCUM, and NCUM+L were evaluated. The data demostrated that the cytotoxicity of each group exhibited dose‐dependent characteristics (Figure 5b). The IC_50_ value of the NCUM group was 1.74 ± 0.46 µg mL^−1^, which was similar to Napabucasin group (Table S4, Supporting Information), implying that encapsulation of Napabucasin in NCUM did not affect its cytotoxicity. The IC50 value of the NCUM+L group was 0.28 ± 0.07 µg mL^−1^, lower than NCUM group (*p* < 0.01), indicating that photodynamic therapy could synergistically kill tumor cells with chemotherapy. These results suggested that NCUM+L has a good antitumor effect in vitro.

Napabucasin binds to STAT3 protein, inhibiting its phosphorylation and thereby suppressing its activity. The Western blot results showed that the content of p‐STAT3 protein in CT26 cells significantly decreased after treatment with Napabucasin, while the total protein content of STAT3 did not show significant changes (Figure 5c; Figure S8, Supporting Information). Tumor stem cell markers, such as Nanog, Sox2, and Oct4, are important molecules that maintain continuous self‐renewal and proliferation of cells. After Napabucasin acts on STAT3 protein, it downregulates the expression of stemness genes driven by STAT3 protein. The expression of tumor stem cell‐related molecules Nanog, Sox2, and Oct4 in CT26 cells were significantly decreased after treatment with Napabucasin (Figure 5d). These results indicate that Napabucasin can inhibit STAT3 protein phosphorylation and reduce the stemness of tumor cells.

### 2.9 Combined Antitumor Effect of FP/NCUM‐Gel

The antitumor effects of FP/NCUM‐Gel was investigated after administrated according to the preset schedule (Figure 5e). During the experimental process, no significant body weight changes was observed (Figure S9, Supporting Information), indicating that FP/NCUM‐Gel has low systemic toxicity. After treatment, the tumor volumes of the CUM and CUM‐Gel groups were lower than NS group (Figure 5f), with tumor inhibition rates of 32.41 ± 9.12% and 41.56 ± 7.16%, respectively (Table S5, Supporting Information), indicating the effectiveness of photodynamic therapy. The tumor volume of the F/CUM‐Gel and FP/CUM‐Gel groups were significantly lower than the CUM‐Gel and F/CUM‐Gel groups, indicating the effectiveness of FLT3L to recruit DC and Poly I:C to activate DC. After the addition of Napabucasin to all treatment groups, tumor volume was significantly reduced (Figure 5h), and the expression of tumor stemness‐related molecules (Nanog, Sox2, Oct4) and tumor stem cell proportion showed a significant decrease (Figure 5j; Figure S10, Supporting Information), indicating that Napabucasin effectively inhibits the STAT3 pathway and reduces tumor cell stemness, enhancing the antitumor effect.

After one treatment cycle, the mice were euthanized and the tumor tissues were separated for *ex vivo* imaging and tumor weight measurement. The results of tumor weight were consistent with those of tumor volume (Figure 5g,i). The NCUM‐Gel group showed significantly lower tumor weight than NCUM group, illustrating that the gel can increase drug efficacy by enhancing drug retention. The FP/NCUM‐Gel group exhibited the best tumor suppression effect, with a tumor inhibition rate of 93.13 ± 1.65%, indicating that inhibiting tumor cell stemness or enhancing DC cell function can significantly enhance the antitumor effect. TUNEL and Ki67 staining were performed on the tumor tissue to detect tumor cell proliferation and apoptosis. The FP/NCUM‐Gel group showed the most strong TUNEL fluorescence and the lowest Ki67 positive rate (Figure 5k,l). Furthermore, the major organs were collected and analyzed by H&E staining for major organ histology. Results showed that compared with the NS group, no damages were observed in FP/NCUM‐Gel group (Figure S11, Supporting Information), indicating the good safety profile of FP/NCUM‐Gel in vivo.

### 2.10 Tumor Immune Microenvironment Improved by FP/NCUM‐Gel

**Figure 6:**
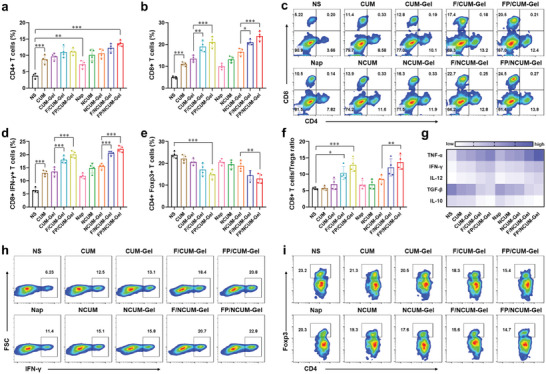
FP/NCUM‐Gel improved the immune microenvironment of tumor tissue after one treatment cycle. a,b) The proportion of CD4^+^ and CD8^+^ T cells in tumor tissue. c) Representative flow diagram of CD4^+^ and CD8^+^ T cells. d) The proportion of CD8^+^IFN‐γ^+^ T cells and e) CD4^+^Foxp3^+^ T cells in tumor tissue. f) The ratio of CD8 + T cells to Tregs. g) ELISA analysis of TNF‐α, IFN‐γ, IL‐12, TGF‐β and IL‐10 in tumor tissues. h) Representative flow diagram of CD8^+^IFN‐γ^+^ T cells and i) CD4^+^Foxp3^+^ T cells. Data were expressed as mean ± SD (*n* = 4).

After FP/NCUM‐Gel treatment, the infiltration of T cells in tumor tissue was promoted to exert an enhanced antitumor immune effect. After one treatment cycle, lymphocytes were isolated from the tumor tissue to analyze the T cell content and evaluate the ability of FP/NCUM‐Gel to improve the tumor immune microenvironment (**Figure** **6**a–c). The flow cytometry analysis results showed that CD4^+^ T cells and CD8^+^ T cells infiltration in CUM and Napabucasin groups were significantly increased. The content of CD3^+^CD8^+^ T cells in FP/NCUM‐Gel group was 23.90 ± 2.08%, which was more abundant than NCUM‐Gel group, illustrating that FLT3L and Poly I:C can promote the infiltration of T cells in tumor tissue through enhanced DC function. Furthermore, analyzing the levels of cytotoxic CTL cells and regulatory Treg cells in the tumor tissue. The results of flow cytometry analysis showed that CTL proportion had a similar trend to CD8^+^ T cells (Figure 6d,h), and Treg proportion had an opposite trend (Figure 6e,i). The FP/NCUM‐Gel group had the highest ratio of CD8^+^ T cells to Treg cells (Figure 6f). These results indicate that FP/NCUM‐Gel can enhance the infiltration of cytotoxic CTL cells and reduce the number of regulatory Treg cells, thus improving the tumor immune microenvironment.

Immune cells regulate the immune microenvironment by secreting cytokines. To evaluate the antitumor immune response, immune‐promoting cytokines tumor necrosis factor alpha (TNF‐α), interferon‐gamma (IFN‐γ), and interleukin‐2 (IL‐2), as well as immune‐suppressive cytokines transforming growth factor beta (TGF‐β) and IL‐10, were measured in tumor tissues. ELISA results showed that the FP/NCUM‐Gel group exhibited the highest levels of TNF‐α, IFN‐γ, and IL‐2, and the minimal TGF‐β and IL‐10 content (Figure 6g). This indicates that FP/NCUM‐Gel can improve immune suppression and activate antitumor immunity.

### 2.11 In Vivo Antitumor Activity on B16F10 Tumor Model

**Figure 7:**
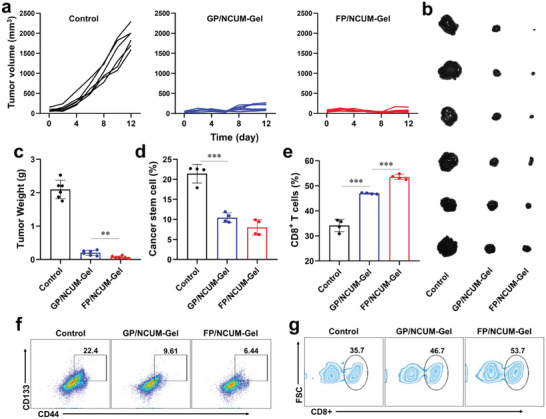
Inhibiting the stemness of tumor cells could further enhance the therapeutic effect of in situ vaccine in B16F10 tumor model. a) Individual tumor volume curves. b) Tumor photos and c) tumor weight at the endpoint. d) Cancer stem cell and e) CD8^+^ T cell proportion in B16F10 tumor tissue. The represent flow diagram of f) cancer stem cells (CD44^+^CD133^+^) and g) CD8^+^ T cells.

To better validate the potential of FP/NCUM‐Gel against tumors, we used B16F10 as the tumor model to re‐evaluate its anti‐tumor effect. The administration regimen was the same as part 2.9. The mice were divided into three groups: Control, GP/NCUM‐Gel (FLT3L replaced with GM‐CSF), and FP/NCUM‐Gel. Tumor volumes and tumor weight after administration showed that GP/NCUM‐Gel and FP/NCUM‐Gel significantly inhibited tumor progression, and in addition, the tumor suppression ability of FP/NCUM‐Gel was significantly stronger than GP/NCUM‐Gel (**Figure** **7**a–c). The proportion of cancer stem cells and CD8^+^ T cells in tumor site were further analyzed. The results showed that both Nap‐containing formulations significantly reduced the proportion of cancer stem cells (Figure 7d,f); FLT3L (FP/NCUM‐Gel) exhibited a stronger potential in inducing CD8^+^ T cell‐mediated immune responses than GM‐CSF (GP/NCUM‐Gel) (Figure 7e,g).

## 3 Conclusion

This study focused on the antigen cross‐presentation mechanism of cDC1 and the mechanism of tumor stemness leading to immune tolerance. We fabricate drug‐loaded temperature‐sensitive hydrogel FP/NCUM‐Gel, which can be gelated in situ to form drug depot after intratumoral injection, achieve deep tumor PDT, and promote in situ tumor antigen release under 980 nm laser irradiation. FLT3L recruits cDC1 to tumor tissue and promotes tumor antigens initialized by cDC1. cDC1 is stimulated to mature by Poly I:C and subsequently migrates to lymph nodes to specifically activate CTLs. Further, napabucasin reduces tumor stemness and assists lymph node‐derived CTLs to achieve effective tumor killing. In summary, we propose a cDC1‐based in situ vaccine strategy that, in combination with tumor stemness inhibition, can potently activate CTLs mediated immune response and diminish tumor immune tolerance, enhancing the efficacy of tumor therapy. This study may provide a new inspiration for the development of effective tumor vaccines.

## Conflict of Interest

The authors declare no conflict of interest.

## Supporting information

Supporting Information

## Data Availability

The data that support the findings of this study are available from the corresponding author upon reasonable request.
