# Supplementary material for: In Situ Hydrogel Modulates cDC1‐Based Antigen Presentation and Cancer Stemness to Enhance Cancer Vaccine Efficiency
Source: Adv Sci (Weinh). 2024 Apr 2;11(20):2305832. doi: 10.1002/advs.202305832 (PMC11132059; doi:10.1002/advs.202305832)
Supplement: Supplementary file 1 — Supporting Information [file ADVS-11-2305832-s001.pdf]

ADVANCED SCIENCE
Open Access

Supporting Information

for *Adv. Sci.*, DOI 10.1002/advs.202305832

In Situ Hydrogel Modulates cDC1-Based Antigen Presentation and Cancer Stemness to Enhance Cancer Vaccine Efficiency

Tong Gao, Shijun Yuan, Shuang Liang, Xinyan Huang, Jinhu Liu, Panpan Gu, Shunli Fu, Na Zhang and Yongjun Liu**

Supporting Information

In situ hydrogel modulates cDC1-based antigen presentation and cancer stemness to enhance cancer vaccine efficiency

Tong Gao‡, Shijun Yuan‡, Shuang Liang, Xinyan Huang, Jinhu Liu, Panpan Gu, Shunli Fu, Na Zhang, and Yongjun Liu**

Experimental Section

Materials: The Napabucasin was purchased from Shanghai MedChemexpress LLC. Chlorin e6 (Ce6), 3-aminopropyltriethoxysilane (APTES), and 1,3-diphenylisobenzofuran (DPBF) were purchased from Shanghai Aladdin Biochemical Technology Co., Ltd. The upconversion nanoparticles (UCNP) were purchased from Xi'an RuiXi Biological Technology Co., Ltd. Hexadecyl trimethyl ammonium bromide (CTAB), tetraethoxysilane (TEOS), and potassium permanganate ($KMnO_4$) were purchased from China National Pharmaceutical Group Chemical Reagent Co., Ltd. Poloxamer F127 and F68, methylthiazol tetrazolium (MTT), and polyinosinic-polycytidylic acid (Poly I:C) were purchased from Sigma-Aldrich Corporation. 2,7-Dichlorodihydrofluorescein diacetate (DCFH-DA), BCA Kit, ATP Kit, and Annexin V-FITC/PI Apoptosis Kit were purchased from Beyotime Biotechnology Co., Ltd. ICD antibodies were purchased from Beijing Biosynthesis Biotechnology Co., Ltd. Mouse HMGB1 ELISA Kit was purchased from Beijing Solarbio Science & Technology Co., Ltd. GM-CSF and FLT3L cytokines were purchased from PeproTech Corporation. Flow cytometry antibodies were purchased from BioLegend. ELISA kits were purchased from Dakewe Biotech Co., Ltd. Western antibodies were purchased from Cell Signaling Technology.

Cell lines and animals: The mouse colon cancer cell line (CT26.WT) was purchased from the Cell Bank of the Chinese Academy of Sciences and cultured in RPMI 1640 cell culture medium (MeilunBio) containing 10% fetal bovine serum and 1% penicillin-streptomycin at 37°C and 5% CO_2. Female Balb/c mice (6-8 weeks old) were purchased from SPF Biotechnology Co.,Ltd. All experiments were performed in accordance with the Regulations for the Administration of Affairs Concerning Experimental Animals of the People's Republic of China and the requirements of the Animal Ethics Committee of Shandong University.

Synthesis and characterizations of Nap-Ce6/UCM (NCUM): Ce6 (2 mg) was dissolved in DMSO (0.5 mL), and EDC and NHS were added to activate the solution under ice-bath conditions. APTES (12 μL) was added to the activated Ce6 solution, and the reaction was

carried out at room temperature for 2 h to obtain APTES-modified Ce6, which was stored in DMSO for subsequent experiments. CTAB (100 mg) was dissolved in deionized water, and UCNP (2 ml, 10 mg/mL) were added and mixed thoroughly. The mixture was placed in a round-bottom flask and stirred overnight to allow complete evaporation of cyclohexane. Deionized water (40 mL), ethanol (6 mL), and NaOH (1 mL, 0.5M) were added to the above solution, and the mixture was stirred at 80°C for 15 min. APTES-modified Ce6 was mixed with TEOS and added dropwise to the above reaction mixture, which was stirred at 80°C for 2 h. The reaction product was then collected by centrifugation at 10000 rpm for 10 min, washed three times with deionized water and ethanol, and the concentration of Ce6 in the supernatant was determined by UV spectrophotometry. The resulting reaction product was dispersed in NH_4NO_3/ethanol solution and refluxed at 65°C for 6 h, repeated three times to remove the surfactant CTAB from the surface, and then centrifuged and washed with deionized water and ethanol to obtain Ce6/UC (CU). Next, under ultrasound conditions, $KMnO_4$ solution (10 mL, 10mg/mL) was dropped into the CU suspension and sonicated for 6 h. The reaction product was collected by centrifugation at 10000 rpm for 10 min, washed three times with deionized water and ethanol to obtain Ce6/UCM (CUM). Napabucasin was loaded into CUM using a passive loading method. CUM (20 mg) was dispersed in a methanol solution of Napabucasin (10 mL, 0.5 mg/mL) under stirring at room temperature for 6 h to allow most of the methanol to evaporate. The product, NCUM, was collected by centrifugation. The concentration of Napabucasin in the supernatant was determined by HPLC.

The morphology of UCNP, CU, and CUM were observed using transmission electron microscopy (TEM). The particle size distribution and zeta potential of UC, CU, and CUM were measured using DLS Zetasizer Nano ZS90. The emission spectrum of CUM was determined using a fluorescence spectrophotometer, while the absorption spectrum of CUM was determined using a UV-visible spectrophotometer. The adsorption isotherms and pore size distributions of CUM and NCUM were determined using a surface area and pore size analyzer. The drug loading (DL) and encapsulation efficiency (EE) of Ce6 and Napabucasin were calculated using the following formulas:

$$\text{DL \%} = (W_{\text{total drug}} - W_{\text{free drug}}) / W_{\text{nanoparticles}} \times 100\%$$

$$\text{EE \%} = (W_{\text{total drug}} - W_{\text{free drug}}) / W_{\text{total drug}} \times 100\%$$

where $W_{\text{total drug}}$ and $W_{\text{free drug}}$ represented the weight of drug in initial solution and free drug in supernatant, respectively. $W_{\text{nanoparticles}}$ represented the weight of NCUM.

Synthesis and characterizations of FL/PIC/NCUM-Gel (FP/NCUM-Gel): Poloxamer F127 and F68 were used as thermosensitive matrices to form a hydrogel at body temperature by

adjusting their mass ratio. Precise amounts of F127 and F68 were weighed and placed in a beaker, followed by the addition of deionized water. The mixture was thoroughly swelled and mixed by placing it in a refrigerator at 4℃, resulting in a clear viscous F127/F68 solution, where F127 concentrations were 16%, 18%, and 20%, and F68 concentrations were 2%, 4%, and 6%. The gelation temperature was used as an evaluation index to optimize the concentrations and ratios of F127 and F68 at an ambient temperature of 25℃. The gelation temperature was determined using the tube inversion method. Specifically, 2 mL of the F127/F68 solution was taken and placed in a tube, which was allowed to equilibrate for 5 min at ambient temperature. Then, the tube was placed in a 25℃ water bath, and the temperature was increased by 1℃/min to 45℃. The gelation temperature was recorded as the temperature at which the inverted tube did not flow.

Using the optimized concentrations and ratios of the gel material, NCUM, FLT3L, and Poly I:C were mixed into the F127/F68 solution to form a uniform mixture. The state of the resulting FP/NCUM-Gel hydrogel under different temperature condition (25°C and 35°C) was photographed. The morphology of the gel was observed using a scanning electron microscope after freeze-drying and gold spraying.

In vitro drug release: The release characteristics of Napabucasin from NCUM was investigated using the dialysis method. NCUM was dispersed in NS, and 1 mL of each sample was placed in a pre-treated dialysis bag (MW=8~14 kDa). The dialysis bags were then placed in 10 mL release medium, which was composed of PBS (pH=6.5), PBS (pH=6.5, containing 1 mM H_2O_2), PBS (pH=7.4), or PBS (pH=7.4, containing 1 mM H_2O_2), all of which contained 1% Tween-80. The samples were shaken in a constant temperature water bath at 37°C, and the release medium was collected at 0.5 h, 1 h, 2 h, 4 h, 6 h, 8 h, 12 h, 24 h, and 48 h, and replaced with fresh medium. The concentration of Napabucasin in each sample point was determined using HPLC, and the cumulative release amount was calculated.

The release characteristics of Napabucasin and model protein IgG from IgG/NCUM-Gel were investigated using the dialysis method. To evaluate the release of Napabucasin, IgG/NCUM-Gel was placed in a pre-treated dialysis bag (MW=8~14 kDa), which was then placed in 10 mL release medium, PBS (pH=6.5, 1 mM H_2O_2, and 1% Tween-80). To evaluate the release of IgG, IgG/NCUM-Gel was placed in a pre-treated dialysis bag (MW=300 kDa), which was then placed in 10 mL of release medium, PBS (pH=6.5). The samples were incubated at 37°C in a constant temperature water bath shaker, and the release medium was collected and replaced with fresh medium at predetermined time points, including 1 d, 2 d, 4 d, 6 d, 8 d, 10 d, 12 d, 14 d, and 16 d. The concentration of Napabucasin at each time point was determined by

HPLC, and the concentration of IgG was measured using an enhanced BCA assay kit. The cumulative release of both Napabucasin and IgG was calculated.

Drug retention and hydrogel degradation: To investigate the *in vivo* gelation and degradation behavior of the hydrogel, Balb/c mice were used. The hydrogel was injected subcutaneously and on 0 d, 7 d, 14 d, and 21 d after administration, mice were euthanized and dissected. The subcutaneous gel morphology was photographed and recorded.

To investigate the *in vivo* retention of the hydrogel, real-time IVIS spectrum system was used. CT26 tumor-bearing Balb/c mice were selected as the animal model, and Cy5.5 was used as a tracer to prepare Cy5.5-Gel. CT26 cell suspension (0.1 mL, 1×10^7/mL) was injected subcutaneously into the right axilla of the mice. When the tumor volume reached about 300 mm^3, free Cy5.5 and Cy5.5-Gel were injected into the tumor. The dose of Cy5.5 was 10 μg per mouse. At 0 d, 1 d, 2 d, 4 d, 6 d, and 8 d after administration, the mice were anesthetized for real-time live imaging. The results were processed using Living Image 3.1.

ROS generation in vivo and in vitro: In order to investigate the ability of NCUM to produce reactive oxygen species (ROS) *in vitro*, 1,3-diphenylisobenzofuran (DPBF) was used. ROS can react with DPBF, causing a decrease in absorbance at 426 nm. CU and CUM were uniformly dispersed in PBS (pH=6.5) with a Ce6 concentration of 2 μg/mL. Dimethylformamide (DMF) solution (60 μL) containing DPBF (0.5 mg/mL) was added to 2 mL PBS, CU solution, and CUM solution, respectively, and mixed well. Under dark conditions, each solution was irradiated with a 980 nm near infrared laser at a power density of 0.5 W/cm^2 for 5 min. The UV absorption spectra of each sample were scanned and the absorbance at 426 nm was recorded at 0 min, 1 min, 2 min, 3 min, 4 min, and 5 min.

To analyze the ability of NCUM to induce ROS production in tumor cells, CT26 cells were seeded in a 24-well plate at a density of 5×10^4 cells/well and incubated overnight. After incubation with ROS fluorescent probe DCFH-DA for 1 h, fresh culture medium containing Napabucasin, CU, CUM, and NCUM, respectively, was added to the cells and incubated for 4 h. Then, the culture medium was replaced with fresh medium, and cells in the CU+L, CUM+L, and NCUM+L groups were irradiated with 980 nm near infrared laser at a power density of 0.5 W/cm^2 for 5 min. DAPI was used to stain the cell nuclei, and fluorescence microscopy was used for imaging. To quantify ROS production, cells were harvested by trypsinization and analyzed using flow cytometry.

In order to investigate the ability of NCUM to induce ROS generation *in vivo* after laser irradiation, CT26 tumor-bearing Balb/c mice were selected as the animal model. CT26 cell suspension (0.1 mL) was injected into the right axilla of each mouse at a density of 1×10^7/mL.

When the tumor volume grew to approximately 300 mm^3, NCUM was injected into the tumor and DCFH-DA was injected 12 h later. Then, the tumor tissue was irradiated with 980 nm near infrared laser at a power density of 0.5 W/cm^2 for 5 min. The mice were euthanized and the tumor tissue was collected for preparation of sections, which were stained with DAPI and imaged using confocal laser microscopy.

Immunogenic cell death in vitro: To evaluate the NCUM induced immunogenic death of tumor cells, CT26 cells were seeded in a 24-well plate at a density of 5×10^4 cells/well overnight. Fresh culture medium containing Napabucasin, CU, CUM, and NCUM was added to the wells and incubated for 4 h. After that, the medium was replaced with fresh culture medium, and the cells in the CU+L, CUM+L, and NCUM+L groups were irradiated with 980 nm near infrared laser at a power density of 0.5 W/cm^2 for 5 min. To analyze the expression of CRT, the cells were incubated for an additional 4 h after laser irradiation, fixed with 4% paraformaldehyde for 10 min, and then incubated with Rabbit anti-mouse CRT primary antibody and Goat anti-Rabbit IgG-AF594 secondary antibody. DAPI was used to label the cell nuclei, and inverted fluorescence microscopy was used for imaging. Flow cytometry was used for quantification. For intracellular HMGB1 detection, the cells were incubated for an additional 12 h after laser irradiation, and then treated with 4% paraformaldehyde and 0.1% Triton X-100. The cells were incubated with Rabbit anti-mouse HMGB1 primary antibody and Goat anti-Rabbit IgG-AF488 secondary antibody, and inverted fluorescence microscopy was used for imaging. To quantify HMGB1 release, the culture medium of the cells was collected, and the level of HMGB1 was measured using an ELISA kit. To analyze ATP secretion, the cells were incubated for an additional 12 h after laser irradiation, and the culture medium of the cells was collected and analyzed using an ATP detection kit.

Vaccine-like effects of ICD: Balb/c mice were chosen as the animal model to investigate the *in vivo* vaccine-like characteristics of tumor cells treated with NCUM+L. Untreated tumor cells were selected as negative controls, while tumor cells treated with classical freeze-thaw cycles were selected as positive controls, the cell suspension was placed in liquid nitrogen and taken out quickly after 5 min and placed in a 37°C water bath until completely thawed, then placed in liquid nitrogen again, and this process was repeated three times. The tumor cell suspension in the experimental group was co-cultured with NCUM and then irradiated with 980 nm near infrared laser at a power density of 0.5 W/cm^2 for 5 min, repeated three times. CT26 cells (1×10^6/group) were pretreated with PBS, freeze-thaw cycles, and NCUM+L, and 12 h later, tumor cells from each group were collected and subcutaneously inoculated into the groin of Balb/c mice. Seven days later, 0.1 mL of normal CT26 cell suspension with a density of

1×10^7/mL was inoculated into the right axilla of the mice, and the growth of the tumor on the right side was observed and recorded.

Induction of GM-DC and FL-DC in vitro: Mononuclear cells were obtained from mouse bone marrow, and DC cells were obtained after induction with different cytokines. Female Balb/c mice aged 6-8 weeks were euthanized under sterile conditions, and their femurs and tibias were harvested and the surrounding muscle tissue was removed. The bone ends were cut with scissors, and bone marrow was flushed out with PBS into a culture dish. The bone marrow suspension was collected, filtered through a 200-mesh nylon mesh to remove bone debris and muscle tissue, and centrifuged at 1200 rpm for 5 min. Then, red blood cell lysis buffer was added at 4°C for 5 min, followed by centrifugation to collect the cells. To obtain GM-DC, cells were cultured in 1640 medium containing GM-CSF (20 ng/ml), IL-4 (10 ng/ml), and 10% fetal bovine serum, at a density of 1×10^6 cells/mL, and the medium with cytokines was half-changed every 2 days. Immature GM-DCs were collected on day 8. To obtain FL-DC, cells were cultured in 1640 medium containing FLT3L (200 ng/ml), GM-CSF (10 ng/ml), and 10% fetal bovine serum, at a density of 1×10^6 cells/mL, and the medium with cytokines was half-changed every 4 days. Immature FL-DCs were collected on day 16. The expression of surface molecules on GM-DCs and FL-DCs, including CD11c, B220, CD103, Clec9, CD11b, Ly6C, and MHC II molecules, were measured by flow cytometry and used for subsequent experiments.

The maturation of GM-DC and FL-DC in vitro: To investigate the ability of NCUM+L-treated tumor cells to promote DC maturation *in vitro*, a Transwell system was used to establish a co-culture system of tumor cells and DCs. GM-DC and FL-DC were seeded in a 12-well plate at a density of 1×10^6 cells/well. CT26 cells were seeded in the Transwell chamber at a density of 1×10^5 cells/well and incubated overnight. Subsequently, fresh medium containing PBS, NCUM, CUM, and NCUM+PIC was added to the chamber and incubated for 4 h. After that, fresh medium was replaced, and the cells in the NCUM group were irradiated with 980 nm near infrared laser at a power density of 0.5 W/cm^2 for 5 min. The Transwell chamber was then placed in a 12-well plate containing seeded DCs and incubated. After 24 h, the DCs and supernatants were collected, and the surface molecules of GM-DC and FL-DC, including CD80, CD86, CD40, CCR7, and MHC I, were analyzed by flow cytometry. The levels of IL-6, IL-12, and TNF-α in the culture medium were detected using ELISA kits.

Recruitment of DC cells in vivo: CT26 tumor-bearing Balb/c mice were used as the animal model to investigate the ability of cytokines to recruit DCs. CT26 cells were injected into the right axilla of the mice at a density of 1×10^7/mL with 0.1 mL of cell suspension. After the tumor volume reached approximately 300 mm^3, NS, GM-CSF, and FLT3L were injected into the

tumor to evaluate their ability to recruit DCs, respectively. Seven days after the injection, the mice were euthanized, and the tumor tissue was collected. The tissue was ground through a copper mesh to obtain a single-cell suspension. The cells were separated using 40% and 60% Percoll solutions to isolate lymphocytes, followed by treatment with blocking solution using rat serum. DCs were labeled using anti-CD11c-APC, anti-CD11b-FITC, and anti-CD103-PE antibodies and analyzed using a flow cytometer. Flowjo software was used to process the results.

In vivo immunization study: To analyze the content of immune cells in lymph nodes, inguinal lymph nodes were isolated and lymphocytes were obtained by grinding and passing through a copper mesh. The lymphocytes were blocked with rat serum and then analyzed for DC cells using anti-CD11c-PE, anti-CD80-FITC, and anti-CD86-PerCP antibodies, and for T cells using anti-CD3-APC, anti-CD4-FITC, and anti-CD8-PE antibodies. Activated T cells were analyzed using anti-CD8-PE and anti-CD69-APC antibodies. The analysis was performed using flow cytometry and the results were processed using Flowjo software. Additionally, lymph nodes were prepared into slices and stained with DAPI to mark the cell nucleus. DC cells were marked with anti-CD11c-PerCP and anti-CD103-APC antibodies, and T cells were marked with anti-CD4-FITC and anti-CD8-PE antibodies. The samples were then imaged using a laser confocal microscope.

To analyze the content of immune cells in tumors, tumor tissues were collected and ground using a copper mesh to obtain a single-cell suspension. The lymphocytes were isolated using 40% and 60% Percoll solutions and treated with rat serum to block nonspecific binding. T cells were analyzed by labeling with anti-CD3-APC, anti-CD4-FITC, and anti-CD8-PE antibodies. For Treg cells, cells were first labeled with external anti-CD4-FITC antibody and subsequently fixed and permeabilized before labeling with internal anti-Foxp3-APC antibody. For CTL cells, protein transport was inhibited by adding 1× Monensin, and then external anti-CD8-PE antibody was labeled followed by fixation and permeabilization before labeling with internal anti-IFN-γ-APC antibody. Flow cytometry was used for cell analysis, and the results were processed using Flowjo software. Additionally, tumor tissues were ground in a 5-fold volume of PBS solution (containing 1% PMSF) using an ultrasonic homogenizer, and the supernatant was collected after centrifugation. The levels of cytokines TNF-α, IFN-γ, IL-12, TGF-β, and IL-10 were measured using an ELISA kit, and the results were processed using ELISACalc software. Furthermore, tumor tissues were prepared into slices and stained with DAPI to mark the cell nucleus. T cells were marked with anti-CD4-FITC and anti-CD8-PE antibodies. The samples were then imaged using a laser confocal microscope.

T cell depletion analysis: CT26 tumor-bearing Balb/c mice were selected to evaluate the antitumor effect of FP/NCUM-Gel after depletion of $CD4^+$ T cells and $CD8^+$ T cells. CT26 cell suspension (0.1 mL, 1×10^7/mL) was injected into the right axilla of the mice. When the tumor volume reached approximately 100 mm^3, the mice were randomly divided into three groups with 6 mice in each group. The $CD4^+$ T cell depletion group received intraperitoneal injection of anti-CD4 antibody, and the $CD8^+$ T cell depletion group received intraperitoneal injection of anti-CD8α antibody, both at a dose of 200 μg/mouse/once every 3 days for a total of 7 times. Meanwhile, all three groups of mice were treated with FP/NCUM-Gel via intratumoral injection, with doses of Napabucasin, Ce6, FLT3L, and Poly I:C at 5 mg/kg, 3 mg/kg, 1.5 mg/kg, and 2.5 mg/kg, respectively. 12 hours after administration, the mice were irradiated with a 980 nm near-infrared laser at a power density of 0.5 W/cm^2 for 5 min on the tumor tissue. The irradiation was performed every 7 days for a total of 3 times. The body weight and tumor length and width of the mice were measured every 2 days, and the tumor volume was calculated. On day 21, the mice were euthanized, and the tumor tissues were dissected for weighing and photographing.

In vitro cytotoxicity: The MTT assay was used to evaluate the cytotoxicity of NCUM on CT26 cells. CT26 cells were seeded in 96-well plates at a density of 5×10^3 cells/well and incubated overnight. To investigate the killing ability of photodynamic therapy on tumor cells, fresh culture medium containing different concentrations of CUM (0.05, 0.5, 5, 10, 25 μg/mL) was added to the wells and incubated for 48 h, and cells in the photodynamic therapy group were irradiated with 980 nm near infrared laser at a power density of 0.5 W/cm^2 for 5 min. To investigate the cytotoxicity of NCUM, Napabucasin and NCUM were added to the wells at gradient concentrations (0.01, 0.1, 1, 2, 5 μg/mL, calculated as Napabucasin) and incubated for 48 h, and cells in the photodynamic therapy group were irradiated with 980 nm near infrared laser at a power density of 0.5 W/cm^2 for 5 min. Subsequently, the MTT reagent was added to the wells, and the absorbance of each well was measured at 570 nm to calculate the cell survival rate.

Cell apoptosis analysis: To investigate the effect of NCUM on tumor cell apoptosis, CT26 cells were seeded in a 12-well plate at a density of 1×10^5 cells/well and incubated overnight. Subsequently, fresh culture medium containing NS, Napabucasin, CU, CUM, and NCUM was added to each well, and the cells were further incubated for 48 h. For the photodynamic therapy groups CU+L, CUM+L, and NCUM+L, the cells were irradiated with 980 nm near infrared laser at a power density of 0.5 W/cm^2 for 5 min. The cells were then collected, incubated with Annexin V-FITC and PI at room temperature in the dark for 20 min, and analyzed using a flow cytometer. The results were processed using Flowjo software.

The stemness of tumor cell: To investigate the ability of NCUM to inhibit STAT3 protein and reduce tumor cell stemness *in vitro*, CT26 cells were seeded into a 12-well plate at a density of 1×10^5 cells per well and incubated overnight. Fresh culture medium containing NS, Napabucasin, and NCUM was added to each well and the cells were incubated for an additional 48 h. Cells in the NCUM+L group were then subjected to photodynamic therapy using 980 nm near infrared laser at a power density of 0.5 W/cm^2 for 5 min. The cells were subsequently collected, and western blot analysis was performed using anti-STAT3 and anti-p-STAT3 antibodies as well as anti-β-Tubulin as the internal reference antibody. Chemiluminescence imaging was conducted using a gel imaging system. Cells subjected to the same treatment were collected, treated with 4% paraformaldehyde and 0.1% Triton X-100, and incubated with Rabbit anti-mouse Nanog, Rabbit anti-mouse Sox2, Rabbit anti-mouse Oct4, and Goat anti-Rabbit IgG-AF594 antibodies. The cells were then quantitatively analyzed using flow cytometry.

CT26 tumor-bearing Balb/c mice were selected as the animal model to evaluate the ability of NCUM to reduce tumor cell stemness *in vivo*. The CT26 cell suspension (0.1 mL, density of 1×10^7/mL) was injected into the right axilla of mice. When the tumor volume grew to approximately 300 mm^3, NS, Napabucasin and NCUM were injected into the tumor, respectively. After 12 h, the mice in the NCUM+L group were irradiated with 980 nm near infrared laser at a power density of 0.5 W/cm^2 for 5 minutes. Mice were euthanized 7 days later, and tumor tissues were harvested for histological analysis. The tissues were incubated with Rabbit anti-mouse Nanog, Rabbit anti-mouse Sox2, Rabbit anti-mouse Oct4 and Goat anti-Rabbit IgG-AF594, and the images were obtained using a laser confocal microscope. Tumor cell suspensions were collected to detect cancer stem cells ($CD44^+CD133^+$).

In vivo antitumor activity on CT26 tumor model: CT26 tumor-bearing Balb/c mice were selected as animal model to evaluate the combination antitumor effect of FP/NCUM-Gel. The mice were inoculated with 0.1 mL of CT26 cell suspension (density 1×10^7/mL) in the right axilla. After the tumor volume grew to approximately 100 mm^3, the mice were randomly divided into ten groups (n=6 per group) and injected intratumorally with 1.NS, 2.CUM, 3.GUM-Gel, 4.FL/CUM-Gel, 5.FP/CUM-Gel, 6.Nap, 7.NCUM, 8.NCUM-Gel, 9.F/NCUM-Gel, and 10.FP/NCUM-Gel. The doses of Napabucasin, Ce6, FLT3L, and Poly I:C were 5 mg/kg, 3 mg/kg, 1.5 mg/kg, and 2.5 mg/kg, respectively. For the photodynamic therapy group, 12 h after administration, the mice were irradiated with 980 nm near infrared laser at a power density of 0.5 W/cm^2 for 5 min, once every 7 days, for a total of 3 times. The mice's weight and tumor size were measured every 2 days, and the tumor volume was calculated. On the 21st day,

the mice were euthanized, and the tumor tissues were dissected, weighed, and photographed. To evaluate the effect of FP/NCUM-Gel on CT26 tumor cell proliferation and apoptosis, the tumor tissues were fixed with 4% paraformaldehyde, embedded in paraffin, and analyzed histologically. The tumor tissues from different treatment groups were observed by Ki67 and TUNEL staining after sectioning.

In vivo antitumor activity on B16F10 tumor model: A B16F10 tumor-bearing C57BL/6J mice model was established to further evaluate the antitumor effect of FP/NCUM-Gel. The mice were divided into 3 groups (1.NS, 2. GP/NCUM-Gel, 3. FP/NCUM-Gel) and treated the same as CT26 tumor model. In group 2, GM-CSF was used to replace FLT3L.

Immunohistochemical analysis: Preliminary safety assessment of the formulation was conducted using the hematoxylin and eosin (H&E) staining method on major organ tissues. After evaluating the combined antitumor effects *in vivo*, major organ tissues, including the heart, liver, spleen, lungs, and kidneys, were dissected, fixed in 4% paraformaldehyde, and embedded in paraffin for histological analysis. The major organ tissues of mice in different treatment groups were sliced and observed by H&E staining.

Statistical Analysis: Statistical analysis was performed using Prism software (GraphPad version 8.0) for Student's t-test and one-way analysis of variance (ANOVA). A significance level of $*p < 0.05$ was considered statistically significant. All data were presented as mean ± standard deviation (SD).

Supplementary Figures and Tables

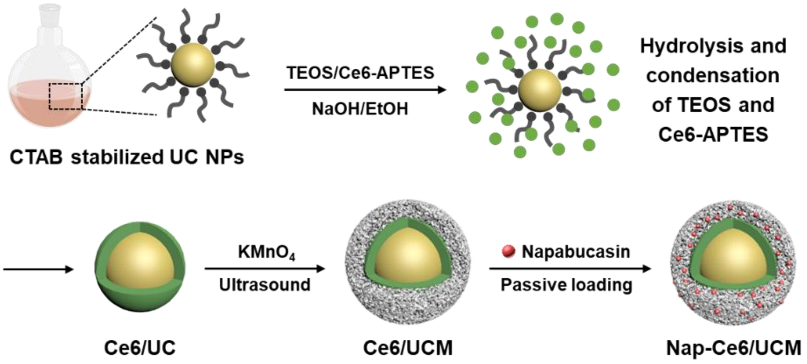

Figure S1. Synthesis of NCUM.

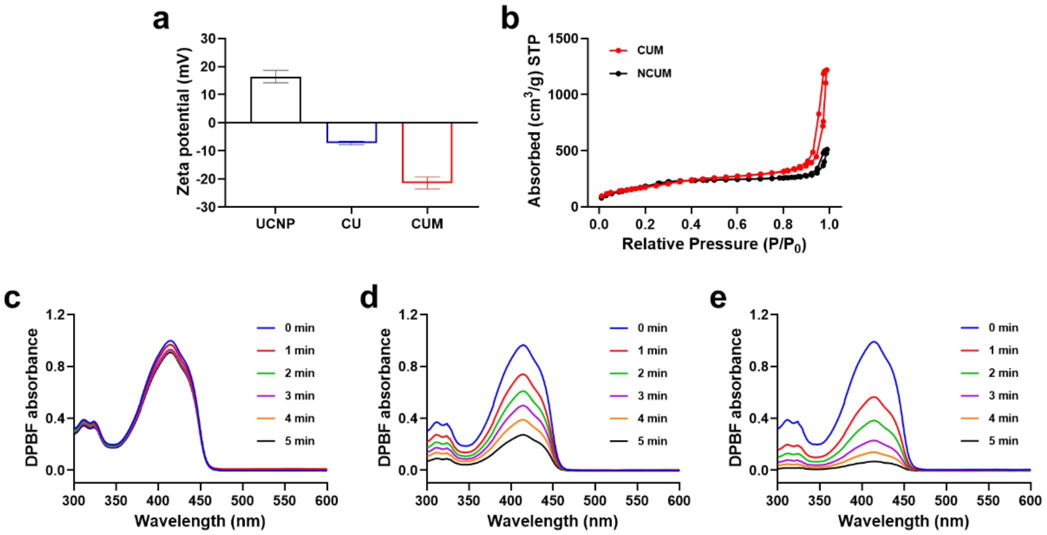

Figure S2. a) Zeta potential of UCNP, CU and CUM. b) Nitrogen adsorption–desorption isotherms of CUM and NCUM. Absorption spectra of DPBF mixed with d) H_2O, e) CU, and f) CUM upon 980 nm laser irradiation for different time durations.

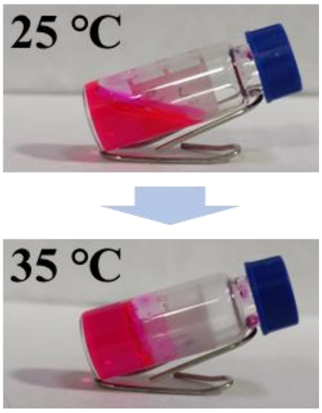

Figure S3. The state of the hydrogel at different temperature conditions (25°C and 35°C).

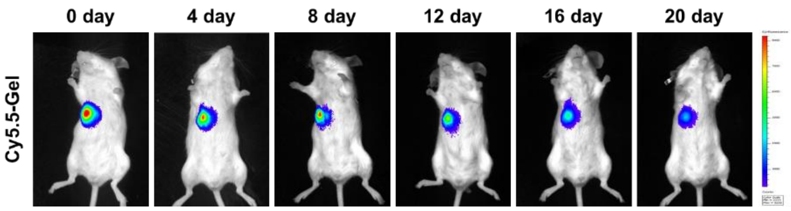

Figure S4. *In vivo* imaging of mice after intra-tumoral injection of Cy5.5-Gel at different time intervals.

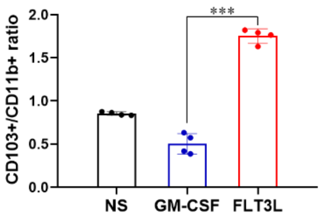

Figure S5. The ratio of $CD103^+$ DC to $CD11b^+$ DC in tumor tissues. Data were shown as mean ± SD (n = 4). ****p* < 0.001.

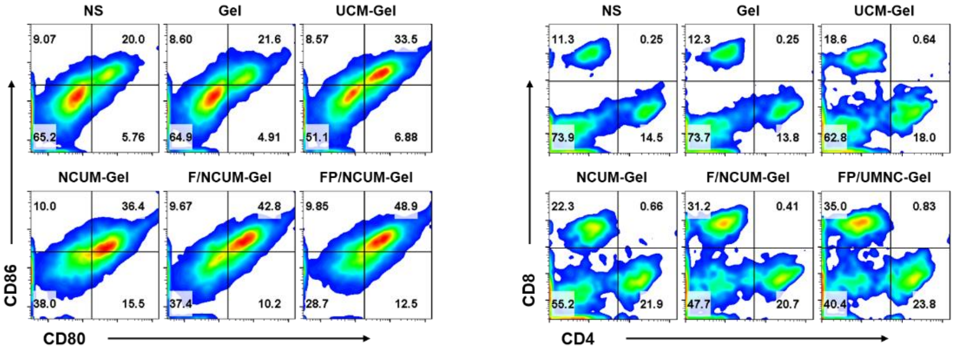

Figure S6 a) Flow cytometry analysis of DCs maturation in lymph nodes (gated on $CD11c^+$ cells). b) Flow cytometry analysis of $CD4^+$ and $CD8^+$ T cells in lymph nodes (gated on $CD3^+$ cells).

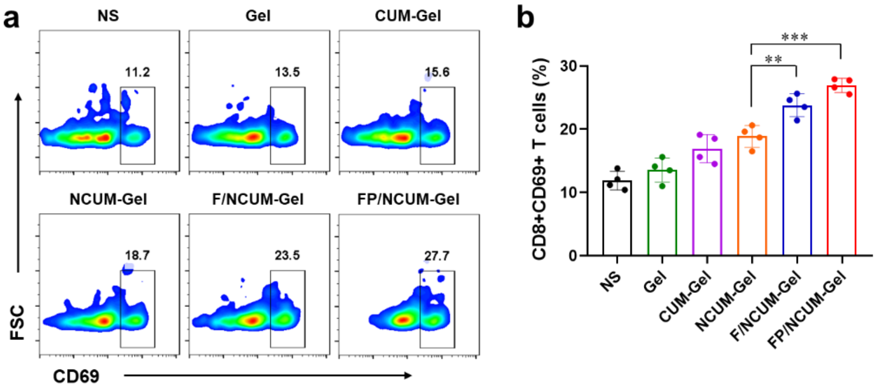

Figure S7. Flow cytometry analysis of $CD8^+CD69^+$ T cells in lymph nodes.

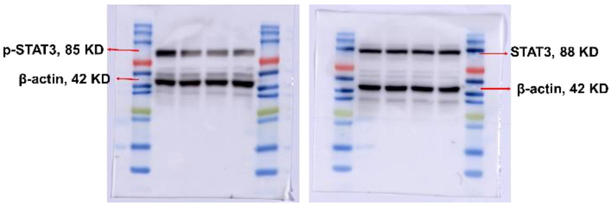

Figure S8. The original image of the western blot results.

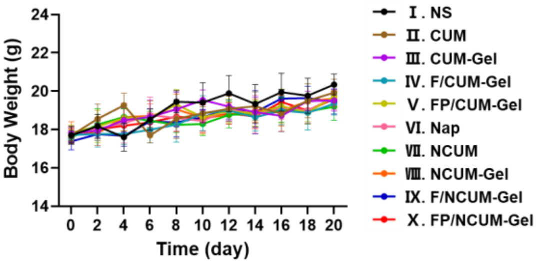

Figure S9. Body weight changes of CT26 tumor-bearing mice after intratumor injection with different formulations. Data were presented as mean ± SD (n = 6).

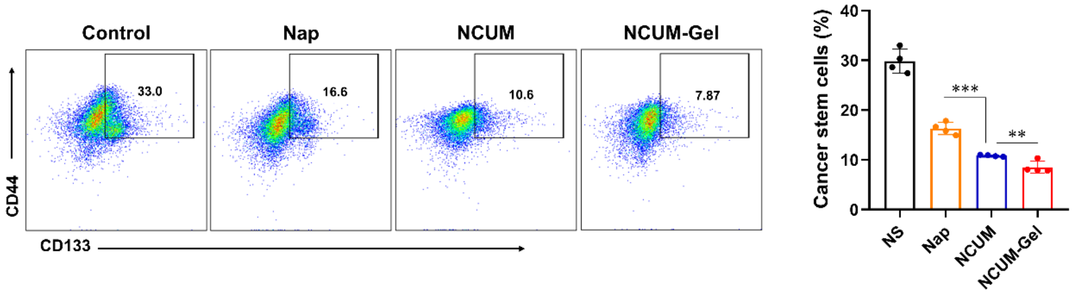

Figure S10. Flow cytometry analysis of cancer stem cells in tumor tissue.

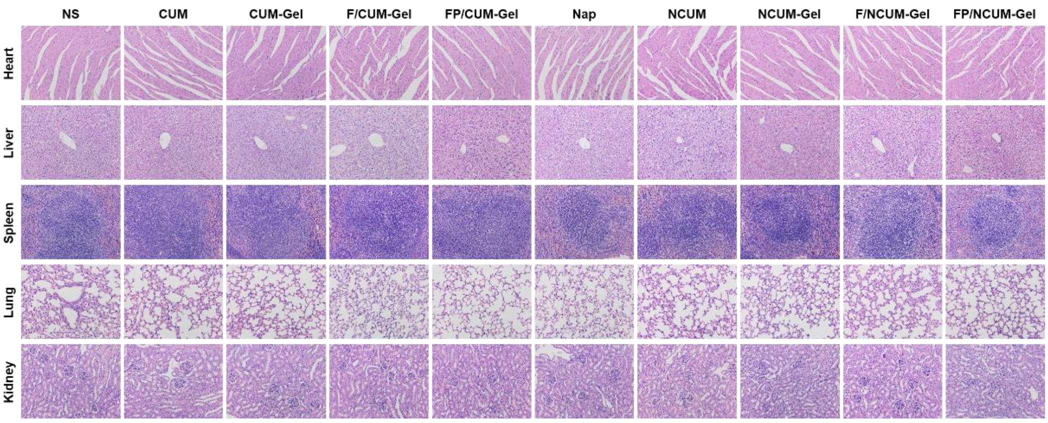

Figure S11. H&E immunohistochemical staining of heart, liver, spleen, lung and kidney after intratumor injection with different formulations. Magnification 200 ×.

Table S1. Particle size and zeta potential of UCNP, CU and CUM. Data were shown as mean ± SD (n=3).

Groups	Size (nm)	PDI	Zeta (mV)
UCNP	53.82 ± 6.97	0.276 ± 0.052	16.47 ± 2.21
CU	67.84 ± 1.13	0.122 ± 0.027	-7.14 ± 0.68
CUM	109.83 ± 7.27	0.113 ± 0.033	-21.37 ± 2.10

Table S2. Drug loading and encapsulation efficiency of Napabucasin and Ce6 in NCUM. Data were shown as mean ± SD (n=3).

Drugs	Drug loading efficiency (%)	Encapsulation efficiency (%)
Ce6	4.06 ± 0.21	87.49 ± 1.11
Nap	9.58 ± 0.57	42.55 ± 3.03

Table S3. Gelling temperature of mixed solution of F127 and F68 with different concentration.

F68	F127		
	16%	18%	20%
2%	34℃	29℃	27℃
4%	38℃	35℃	32℃
6%	40℃	38℃	35℃

Table S4. The IC50 values of Napabucasin, NCUM and NCUM+L on CT26 cells in *vitro*. Data were shown as mean ± SD (n=3). ***p* < 0.01, compared with NCUM group.

Groups	IC50 (μg/mL)
Nap	1.02 ± 0.48
NCUM	1.74 ± 0.46
NCUM+L	0.28 ± 0.07**

Table S5. Tumor inhibition rate of tumor-bearing mice after intratumor injection with different groups. Data were shown as mean ± SD (n=6).

Groups	Tumor inhibition rate (%)
CUM	32.41 ± 9.12
CUM-Gel	41.56 ± 7.16
F/CUM-Gel	65.62 ± 6.18
FP/CUM-Gel	79.22 ± 6.20
Nap	46.39 ± 8.65
NCUM	62.79 ± 5.05
NCUM-Gel	75.72 ± 3.99
F/NCUM-Gel	87.10 ± 2.31
FP/NCUM-Gel	93.13 ± 1.65
